# An inverse causal association between genetically predicted vitamin D and chronic obstructive pulmonary disease risk

**DOI:** 10.3389/fnut.2023.1111950

**Published:** 2023-03-15

**Authors:** Kening Lu, Jiang-Shan Tan, Tian-Qi Li, Jiaqin Yuan, Han Wang, Wenting Wang

**Affiliations:** ^1^State Key Laboratory of Crop Genetics and Germplasm Enhancement, Cotton Hybrid R&D Engineering Center (Ministry of Education), College of Agriculture, Nanjing Agricultural University, Nanjing, Jiangsu, China; ^2^Emergency Center, National Center for Cardiovascular Diseases, Fuwai Hospital, Chinese Academy of Medical Sciences and Peking Union Medical College, Beijing, China; ^3^Key Laboratory of Pulmonary Vascular Medicine, State Key Laboratory of Cardiovascular Disease, National Center for Cardiovascular Diseases, Fuwai Hospital, Chinese Academy of Medical Sciences and Peking Union Medical College, Beijing, China; ^4^Department of Orthopedics, The Second People’s Hospital of Yibin, Yibin, China; ^5^Department of Cardiology, The Third People's Hospital of Chengdu, Affiliated Hospital of Southwest Jiaotong University, Chengdu, Sichuan, China; ^6^Department of Anesthesiology, The Second Affiliated Hospital of Hainan Medical University, Haikou, China

**Keywords:** GWAS, COPD, vitamin D, MR, two-sample

## Abstract

**Aim:**

Observational studies have reported that levels of vitamin D were associated with the incidence of chronic obstructive pulmonary disease (COPD), but the relationship between them may have been confounded in previous studies. In this study, we aimed to determine the relationship between the levels of 25-hydroxyvitamin D (25OHD) and the risk of COPD by two-sample Mendelian randomization (MR) analysis.

**Methods:**

Summary statistics for 25OHD and COPD in this study were obtained from the EBI (*n* = 496,946) consortium and Finn (*n* = 187,754) consortium. MR was adopted to explore the effect of the genetically predicted levels of 25OHD on the risk of COPD. Based on three assumptions of MR analysis, inverse variance weighting was used as the main analysis. To make our results more robust and reliable, MR Egger’s intercept test, Cochran’s Q test, funnel plot, and “leave-one-out” sensitivity analysis were used to assess the potential pleiotropy and heterogeneity in this study. Then, colocalization analysis and MR Steiger approaches were used to estimate the possible directions of estimates between them. Finally, we analyzed the causal associations between the four core genes (DHCR7, GC, CYP2R1, and CYP24A1) of vitamin D and the levels of 25OHD or the risk of COPD.

**Results:**

Our results showed that each 1 standard deviation (SD) increase in the genetically predicted 25OHD level was associated with a 57.2% lower relative risk of COPD [odds ratio (OR): 0.428, 95% Cl: 0.279–0.657, *p* = 1.041 × 10^−4^], and the above association was also verified by maximum likelihood (OR: 0.427, 95% Cl: 0.277–0.657, *p* = 1.084 × 10^−4^), MR–Egger (OR: 0.271, 95% CI: 0.176–0.416, *p* = 2.466 × 10^−4^), MR-PRESSO (OR: 0.428, 95% Cl: 0.281–0.652, *p* = 1.421 × 10^−4^) and MR-RAPS (OR: 0.457, 95% Cl: 0.293–0.712, *p* = 5.450 × 10^−4^). Furthermore, colocalization analyses (rs3829251, PP.H4 = 0.99) and MR Steiger (“TRUE”) also showed a reverse association between them. Besides, the core genes of vitamin D also showed similar results except for CYP24A1.

**Conclusion:**

Our findings provide evidence for a reverse association between genetically predicted 25OHD levels and COPD risk. Taking measures to supplement 25OHD may help reduce the incidence of COPD.

## 1. Introduction

Chronic obstructive pulmonary disease (COPD) is a common and currently treatable but incurable disease. The World Health Organization estimates that COPD will be the third leading cause of death globally by 2030, but that prediction has already come true ahead of time, causing 3.23 million global deaths in 2019, with a significant social and economic burden (1). The disease is a progressive disorder characterized by persistent airflow restriction due to structural changes and chronic inflammation (2). Patients with COPD experience a progressive decline in lung function, a decreased capacity for exercise, frequent disease exasperations, and the progress of extrapulmonary comorbidities such as cardiovascular disease, osteoporosis, and infections (3). COPD poses a serious threat to the lives and health of patients. In addition, it also makes a huge economic burden on society and families because of the high cost of treatment despite great progress has been made in understanding its pathogenesis, natural history, and management, there still exist many open questions, especially concerning how to exactly control the risk factors.

25-Hydroxyvitamin D (25OHD) can best reflect human vitamin D status. A correlation has been demonstrated between 25OHD levels and the status of lung function, including COPD. For example, an age-matched controlled study reported that the patients in the COPD group had significantly lower vitamin D levels than those without the COPD group, suggesting that COPD patients have a higher vitamin D deficiency risk (4, 5). Jorde et al. (6) in a cross-sectional observational study found that there was a fair correlation between serum 25OHD and the current FEV1. Vitamin D-deficient patients had a greater FEV1 decrease than the others in the Norway cohort (7). A prospective cohort study reported an association of lower plasma 25OHD levels with lower lung function, a faster decline in lung function, and a higher risk of COPD (8). At present, the mechanism of how vitamin D reduces the risk of COPD is not clear. Some researchers have attempted to explain their relationship in terms of various immunomodulatory effects and airway smooth muscle remodeling (9). However, most of the current reports are based on observational studies, which are vulnerable to reverse associations, confounding factors, or limited sample sizes.

Mendelian randomization (MR) is a genetic instrumental variable analysis in which observational data can be applied to reconfirm the correlation between genetically predicted exposure (25 OHD) and outcome (COPD) by using genetic variation associated with different levels of modifiable risk factors to assess the impact of risk factors on disease. Furthermore, MR study designs are less susceptible to confounding and reverse bias than observational studies because genetic variation will not be affected by disease state or other risks (10).

Building on the previous genome-wide association study (GWAS), which was finished based on real detected 25OHD levels, we predicted the 25OHD levels based on our selected significant SNP loci. And then, the relationship between genetically predicted 25OHD levels and COPD was further explored by using a two-sample MR analysis in the present study.

## 2. Methods

### 2.1. Study design

**Figure 1:**
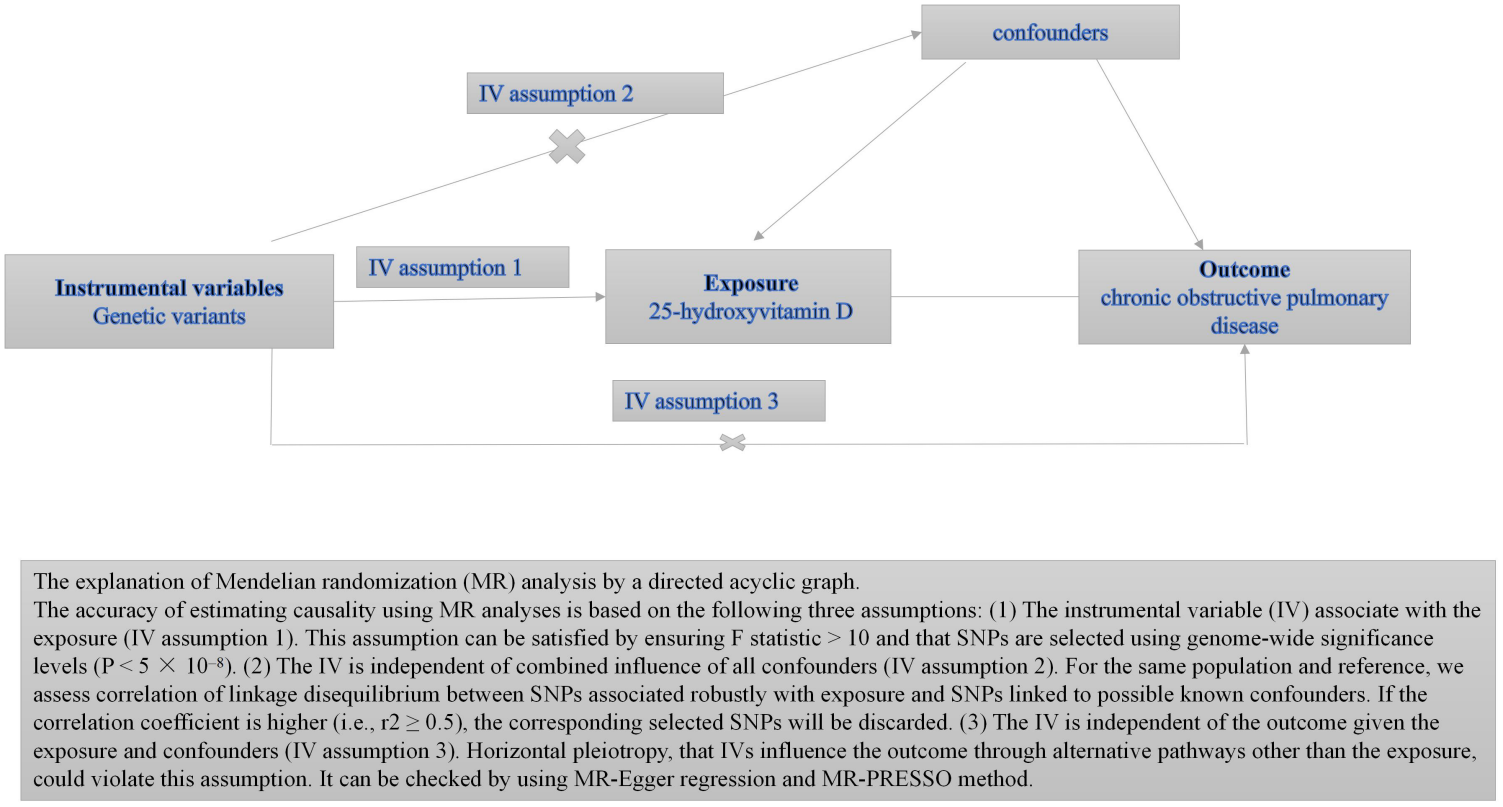
Directed acyclic graphs for the classical Mendelian randomization designs.

In this study, we conducted a two-sample MR approach (Figure 1) (11).

### 2.2. Data sources

#### 2.2.1. 25-hydroxyvitamin D

To evaluate the causal correlation between genetically predicted 25OHD and COPD risk, single-nucleotide polymorphisms (SNPs) as instrumental variables (IVs) were acquired from the EBI consortium, which was a large GWAS (12). This GWAS identified 496,946 individuals and 103 IVs for 25OHD by performing a genetic principal component cluster analysis (13). In their original GWAS, they found that 103 SNPs were significantly associated with the serum 25OHD levels, which were measured in the actual work. Therefore, we used these SNPs to investigate the correlation between genetically predicted serum 25OHD levels and the risk of COPD by using two-sample MR (14, 15).

#### 2.2.2. Chronic obstructive pulmonary disease

Summary-level genetics for COPD were acquired from the FinnGen biobank (Finn) consortium, including 1,031 COPD patients and 186,723 controls from European ancestry, which was acquirable at: https://gwas.mrcieu.ac.uk/datasets/finn-b-COPD_INSUFFICIENCY/.

### 2.3. Genetic instrumental variables

We selected IVs that satisfy the following standard: (1) SNPs highly associated with vitamin D had genome-wide significance (*p* < 5 × 10^−8^) (2). To avoid linkage disequilibrium (LD) caused by vitamin D-related SNPs, LD must satisfy *r*^2^ < 0.001, window size = 10,000 kb, and minor allele frequency > 0.01. LD levels were assessed from the 1,000 Genomes Project with European samples (3). To guarantee a strong relationship between IVs and 25OHD, we chose an *F*-statistic > 10 to avoid bias caused by weak IVs (4, 16, 17). To ensure the second MR hypothesis that genetic variants are independent of potential confounders, we conducted a query in the PhenoScanner database to exclude the contained IVs associated with known confounders. Finally, genes near each IV were extracted and marked for their functions (18).

### 2.4. Mendelian randomization estimates

In this two-sample MR design, we used inverse variance weighting (IVW), weighted median, MR–Egger, maximum likelihood, MR pleiotropic residual sum, and outlier test (MR-PRESSO) and MR using robust adjusted profile score (MR-RAPS) analyses (19–22).

First, we performed a preliminary analysis adopting the IVW method to detect the correlation between genetically predicted 25OHD and COPD. Second, to avoid potential weak IVs, horizontal pleiotropy, and sample overlap issues affecting this MR result, we further verified the relationship by using MR pleiotropy residual sum and outlier test (MR-PRESSO), MR-RAPS, Weighted median, MR Egger. MR–Egger was based on the assumption of InSIDE to conduct a weighted linear regression of exposure results, but it is vulnerable to IVs. Weighted median can significantly improve the detection ability of effects and decrease type I errors. Compared with the weighted median, MR–Egger, MR-RAPS is a new approach of MR analysis that accounts for uncorrelated and correlated pleiotropy and can reduce false positives and increase power. Third, there is generally no bias of weak IVs if the *F* statistic > 10, *F* statistics = (β/SE)^2^ (23). Finally, the corresponding odds ratio (OR) and 95% confidence interval (CI) are calculated to interpret the MR results.

Furthermore, we also tested whether genetically predicted 25OHD and COPD shared a variant in a specific region using symbiosis analysis with PP.H4 values > 0.75 were considered a mark of symbiosis (24). In addition, we adopted the MR Steiger approach to estimate the orientation on exposure and outcome of each extracted IV (25). The result of “TRUE” means predicting association in the expected orientation.

Besides, to further verify the reliability of the results, we used the four core genes of vitamin D for further analysis.

### 2.5. Pleiotropy and heterogeneity analysis

We applied different approaches to explore potential effects to ensure that IVs were independent of outcomes except for exposure. First, we adopted the funnel plot methods, leave-one-out sensitivity analysis, and Cochran Q statistic to analyze the heterogeneity of the contained IVs (26). Second, we used MR–Egger regression to account for horizontal pleiotropy. Finally, we apply global, outlier, and distortion tests as additional controls for pleiotropy adopting the MR-PRESSO R package.

All statistical analyses were conducted by R software (version 4.1.2), The Two-Sample MR (version 0.5.6), and MRPRESSO packages. *p* < 0.05 were considered statistically significant.

## 3. Results

### 3.1. Genetic IVs for 25-hydroxyvitamin D

In this MR design, 103 LD-independent IVs that were significantly associated with 25OHD were included after the clumping process, with 2.70% of the variance explained by the included genetic instrument. Furthermore, the *F* values of all included IVs were greater than 30.45, the influence of weak IVs was effectively eliminated on MR analysis and the results were relatively stable (Supplementary Table S1).

### 3.2. MR analysis for The potential association

In IVW, each 1 SD in 25OHD level was associated with a 57.2% lower relative risk of COPD (OR: 0.428, 95% Cl: 0.279–0.657, *p* = 1.041 × 10^−4^).

**Figure 2:**
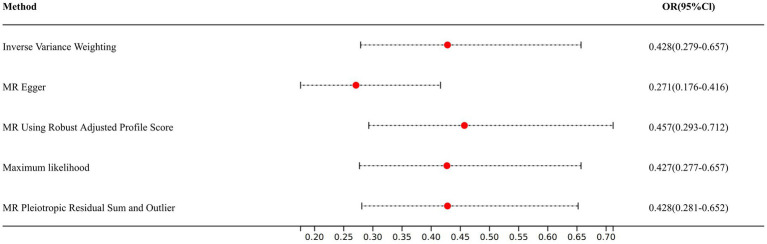
Association of Vitamin D with chronic obstructive pulmonary disease in two-sample Mendelian randomization.

In addition, we found similar results in the other sensitivity analyses, such as MR–Egger (OR: 0.271, 95% CI: 0.176–0.416, *p* = 2.466 × 10^−4^), the maximum likelihood method (OR: 0.427, 95% Cl: 0.277–0.657, *p* = 1.084 × 10^−4^), the MR-RAPS method (OR: 0.457, 95% Cl: 0.293–0.712, *p* = 5.450 × 10^−4^), and the MR-PRESSO method (OR: 0.428, 95% Cl: 0.281–0.652, *p* = 1.421 × 10^−4^; Figure 2).

### 3.3. Heterogeneity and horizontal pleiotropy analysis

**Figure 3:**
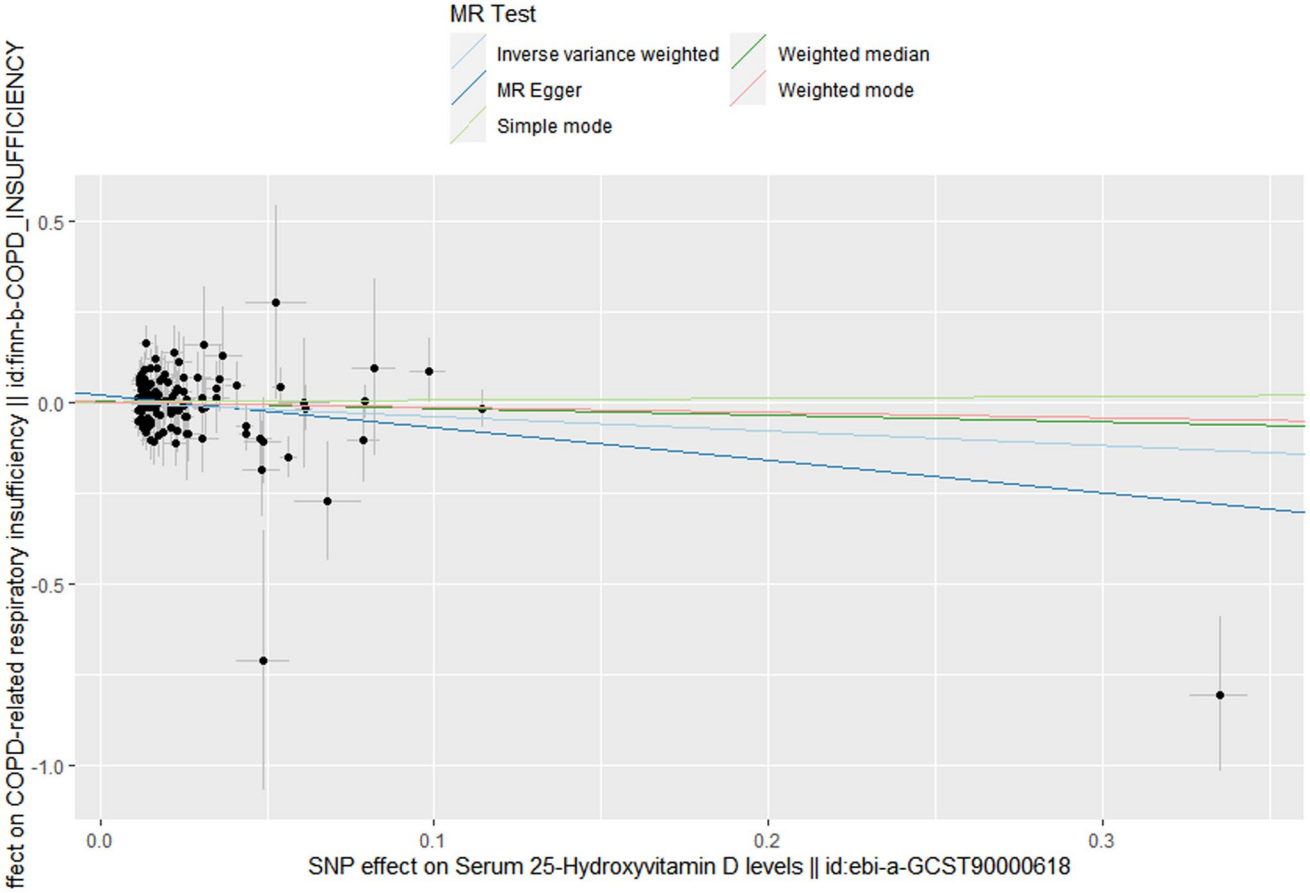
Scatter plot to visualize causal effect of Vitamin D on the risk of chronic obstructive pulmonary disease.

**Figure 4:**
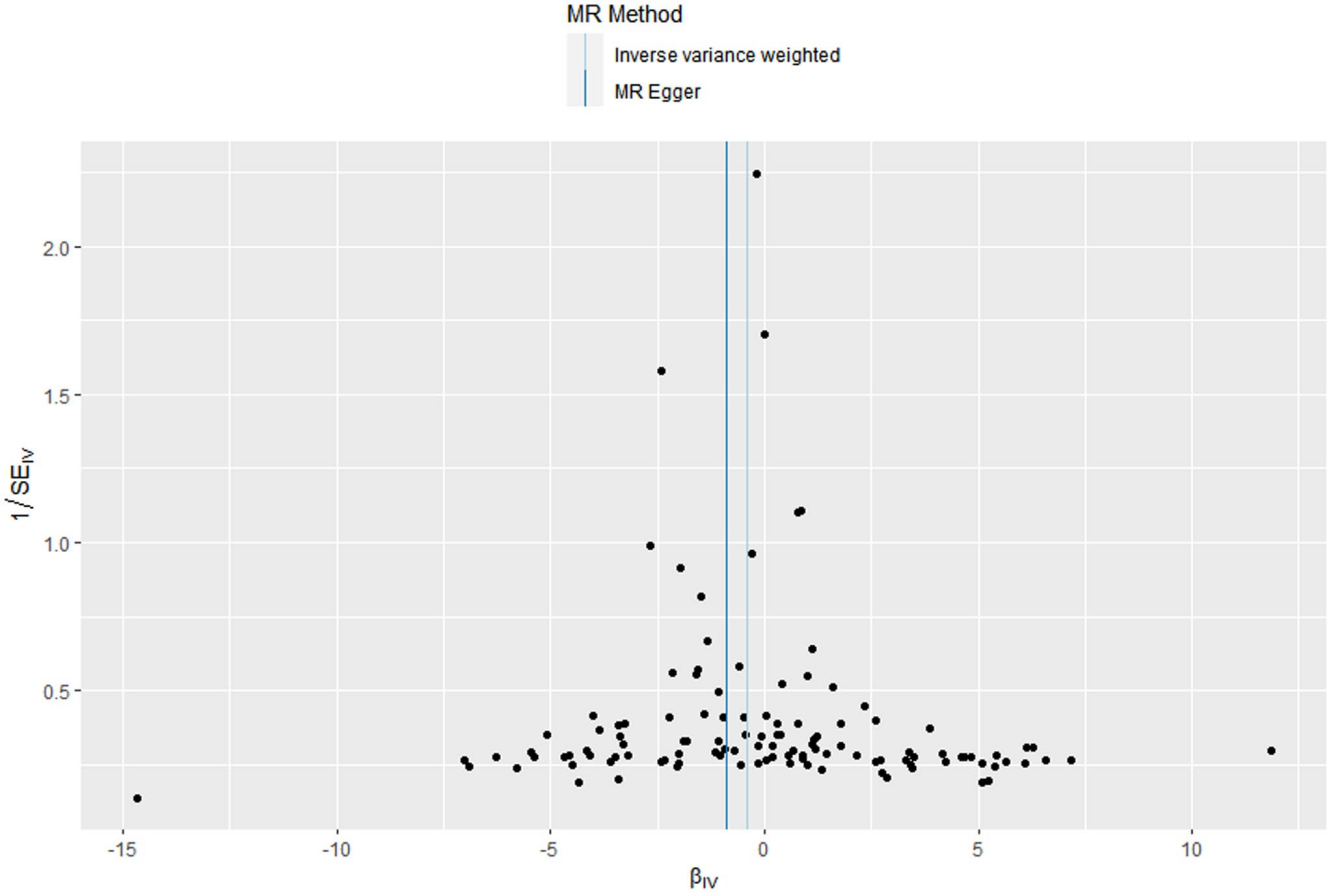
Funnel plots to visualize overall heterogeneity of Mendelian randomization (MR) estimates for the effect of Vitamin D on the risk of chronic obstructive pulmonary disease.

In this MR design, we adopted a range of approaches to estimate the heterogeneity and horizontal pleiotropy in our studies. Cochran’s Q test reported that there was no heterogeneity between genetically predicted 25OHD and COPD (*p* = 0.586). The MR–Egger intercept test reported that there was no horizontal pleiotropy in this study (*p* = 0.087). The “leave-one-out” sensitivity analysis reported that the IVs contained in our study had no significant effect on the results, indicating significant confidence in the results (Figure 3). The funnel plot reported a single asymmetric distribution of IVs, suggesting that the correlation is unlikely to be influenced by potential bias (Figure 4). The results of the MR-PRESSO analysis were consistent with those of IVW, showing that there were no strange outliers in the IV genes contained in our study.

### 3.4. Colocalization analyses and MR Steiger

Our results showed that genomic test regions included a shared variant for 25OHD and COPD (rs3829251, PP.H4 = 0.99). Moreover, using the MR Steiger approach, we also observed a reverse association between genetically predicted 25OHD-related IVs and COPD risk.

### 3.5. A further examination of the four core genes involved in the process of vitamin D

In the further analysis, we found that only three genes could be found in the “ieu open GWAS project,” including DHCR7, CYP2R1, and CYP24A1. As is shown in Table 1, all of the three core genes showed similar results with the main analysis except for CYP24A1.

**Table 1:** The effect of core genes involved in the process of vitamin D.

Core genes	ENSG ID	Traits ID	β of 25OHD on core gene	β of core gene on COPD
DHCR7	ENSG00000172893	eqtl-a-ENSG00000172893	0.09	−0.40
CYP2R1	ENSG00000186104	eqtl-a-ENSG00000186104	0.28	−1.47
CYP24A1	ENSG00000019186	eqtl-a-ENSG00000019186	0.04	0.07

## 4. Discussion

Our study assessed the potential role of vitamin D levels on COPD risk by using a new genetic tool (two-sample MR design). The results showed that the COPD risk decreased as genetically predicted 25OHD levels increased, suggesting that vitamin D levels may play a key role in the occurrence and development of COPD.

Recently, there have been growing studies exploring the potential noncalcifying role of vitamin D and the association between vitamin D and chronic diseases, especially COPD. In prior researches, the correlation between vitamin D and the risk of COPD has been explored. Many observational studies have reported the complex correlations among the serum 25-OHD levels, systemic inflammation, disease severity, and progression (6, 7, 27). A prospective cohort study with 10,116 participants from the Copenhagen City Heart Study and 8,391 participants from the Copenhagen General Population Study also reported a correlation between lower plasma 25OHD levels with lower lung function, a faster decline in lung function, and a higher risk of COPD (HR: 1.58, 95% CI: 1.05–2.40 and HR: 2.00, 95% CI: 1.19–3.36 for the two studies, respectively) (8). In the present study, we found a reverse relationship between genetically predicted 25OHD and COPD, with the relative risk of COPD decreasing with genetically predicted increasing 25OHD concentrations, which is consistent with previous observational findings. Even though we used the significant SNPs rather than the actual measured serum 25OHD levels, we can explore the association, rather than the observational association due to that these SNPs have been randomly assigned to the offspring before the disease occurred and few confounders can bias the conclusion of MR analysis, which is the most significant strength of MR analysis.

In the present MR, we only explored the association between genetically predicted increasing 25OHD concentrations and COPD risk. Therefore, whether patients with COPD may benefit from the supplementation with vitamin D cannot be further analyzed in our study. However, some other research found that timely supplementation with vitamin D can delay the course of COPD patients and improve their lung function. For example, Lehouck et al. (28) reported that 30 participants with severe vitamin D deficiency at baseline (serum 25OHD level of 10 ng/ml) showed a significant reduction in exacerbations in patients with a timely supplementation of vitamin D compared to the placebo group. Besides, Martineau et al. (29) also indicated that the relationship between vitamin D deficiency in COPD patients reduced the risk of moderate or severe exacerbations.

Multiple mechanisms may contribute to the relationship between vitamin D and COPD. First, animal studies showed that vitamin D had positive effects on fibroblast proliferation, alveolar type II cells, alveolarization, and surfactant synthesis, and it may improve the lung function of patients and reduce the risk of CPOD (30). Second, immunological studies found evidence for Th1 immunity and Th17 involvement in COPD (5). For example, vitamin D deficiency may enhance inflammation, chemokine production *via* NF-ƘB, parenchymal degradation, TNF-α, IL-18, histone acetylation, corticosteroid resistance, and matrix metalloproteinase (MMP)-9 production. Third, vitamin D also plays a role in remodeling airway smooth muscle to regulate lung function in COPD (31). Besides, increasing 25OHD concentrations in COPD patients to the optimal range may reduce accompanying exacerbations and bacterial load (32–37).

Ahn et al. (38) reported a significant relationship between rs3829251 and 25OHD (*p* = 8.8 × 10^−7^), and our colocalization analyses inferred that rs3829251, which is located in the 7-dehydrocholesterol reductase gene, might impact the concentration of human 25OHD by regulating the expression of NADSYN1/DHCR7, consequently reducing COPD risk. However, how the rs3829251 locus affects the occurrence of COPD by impacting 25OHD is currently unclear. Thus, more research is needed in the future.

A strength of this study is that we used an MR design to explore the relationship between vitamin D and the COPD risk by using summary data from the largest GWAS studies, which can minimize the residual confounding and possibly reversed bias compared to previous observational studies. Furthermore, we further validate our results by applying other approaches, such as MR-PARS, MR-PRESSO and maximum likelihood, and the consistency of the results makes our results more robust. In addition, we also adopted colocalization analyses and MR Steiger approaches to make our results more reliable.

However, some limitations in this two-sample MR study should be noted. First, the GWAS dataset used in this study is based on a population of European ancestry, and it is unclear whether the results apply to people of non-European ancestry. Second, we cannot completely rule out the possible interactions of gene–environment or diet–gene that influenced our results. In addition, it is difficult for us to evaluate whether Vitamin D supplementation influenced the rate of moderate or severe COPD exacerbations due to the original GWAS involving all the patients, including both moderate and severe COPD exacerbations. Finally, sensitivity analyses such as weighted median, MR–Egger, MR-PRESSO, etc., were used to test and correct the potential horizontal pleiotropy; however, we were unable to address the issue of unobserved pleiotropy, which may affect the conclusion of evaluating the association between genetically predicted vitamin D and the risk of COPD.

## 5. Conclusion

Our study showed that vitamin D levels may be negatively associated with COPD risk. Further studies are needed to elucidate the underlying mechanism.

## Data availability statement

The datasets presented in this study can be found in online repositories. The names of the repository/repositories and accession number (s) can be found in the article/Supplementary material.

## Author contributions

KL and J-ST designed the study and wrote the manuscript. KL, J-ST, T-QL, JY, and HW contributed to the data analysis and data interpretation. WW contributed to the revision of the manuscript. All authors contributed to the article and approved the submitted version.

## Funding

This work was supported by the National Natural Science Foundation of China Grant Awards (grant number 81960669) and the science and technology project of Guozhong Healthcare (grant number GGH-SDEPCH-HP-20230101).

## Conflict of interest

The authors declare that the research was conducted in the absence of any commercial or financial relationships that could be construed as a potential conflict of interest.

## Publisher’s note

All claims expressed in this article are solely those of the authors and do not necessarily represent those of their affiliated organizations, or those of the publisher, the editors and the reviewers. Any product that may be evaluated in this article, or claim that may be made by its manufacturer, is not guaranteed or endorsed by the publisher.
